# Perceived benefits and health risks of cigarette smoking among young adults: insights from a cross-sectional study

**DOI:** 10.1186/s12971-015-0044-9

**Published:** 2015-07-31

**Authors:** Umesh Raj Aryal, Dharma Nand Bhatta

**Affiliations:** Department of Community Medicine, Kathmandu Medical College, Sinamangal, Kathmandu, Nepal; Department of Public Health, Pokhara University, Nobel College, Sinamangal, Kathmandu, Nepal; Faculty of Medicine, Epidemiology Unit, Prince of Songkla University, Hatyai, Thailand

**Keywords:** Current smoking, College students, Perceived health risks, Perceived benefits, Hazard ratio

## Abstract

**Background:**

Perceptions of smoking-related health risks and benefits among young adults (18–24 years) and their smoking behaviour have not been adequately studied in low-income countries like Nepal. This study has examined the perceived risks and the benefits of smoking among young adults who smoke vs. don’t smoke.

**Methods:**

A cross-sectional study was carried out from August to September 2013 among 315 young adults (18–24) from four conveniently selected private colleges of different faculties in Kathmandu Metropolis. The anonymous, self-administrated and semi structured questionnaire contained the information on individual information; smoking behaviour; and perceptions on smoking-related risks and benefits. Kaplan-Meier analysis was used to identify the mean age of smoking initiation. Cox proportion hazard regression was used to assess the relationship between current smoking behaviours and the perceived risks and the benefits of smoking.

**Results:**

Overall, the prevalence of current smoking was 16.2 % (Male =28.4 % and female =5.38 %). The mean age of smoking initiation was 16.6 and 17.7 years for male and female respectively. The risk of becoming a current smoking being a management student was higher (HR = 4.72, 95 % CI: 2.19; 10.20) than being a medical student. The risk of current smoking behaviour significantly increased with those who believed that smoking was enjoyable (HR = 4.74, 2.58; 8.72); would help to deal with problems or stress (3.19, 1.76; 5.79); would feel comfortable with friends (4.29, 2.33; 7.92); would be relaxing (6.95, 3.60; 13.43); and something to do when feel bored (3.42, 1.91; 6.13). The young adults who believed that smoking would make yellow teeth (0.53, 0.30; 0.94) and yellow nail (0.53, 0.29; 0.95); and would be bad to their health (0.45, 0.21; 0.98) were significantly at lower risk of becoming a current smoking.

**Conclusion:**

Positive perceptions related to smoking are common among young adults. To discourage smoking, future intervention programs should focus communicating not only health risks but also counteract perception of benefits related to smoking.

## Background

Tobacco smoking is an important risk factor accountable for non-communicable diseases (NCDs) such as cancer, cardiovascular diseases, diabetics, and chronic respiratory diseases [1]. In Nepal, NCDs are accountable for 50.2 % of total deaths of which 50.7 % were female [2]. WHO STEP Survey 2013 revealed the prevalence of current daily smoking was 18.5 % and their mean age of smoking initiation was 18.2 years (men = 18.5 years; women = 17.6 years) [3]. Next, the mean age of smoking initiation was 16 years among 15–29 years old population [3]. Recent global health professional school survey reported that 18 % of Nepalese medical students were smokers, of them 28 % were male [4]. Another study from Nepal revealed that 16.8 % of public health students were smokers [5]. Similarly, a study from Pokhara, Nepal showed nearly 55 % of medical students were current smokers [6]. These findings revealed that college students or young adults (18–24 years) were vulnerable to cigarette smoking, for young adults, in this phase of life, were exposed to several psycho-social risk factors including the perceived risks and benefits of cigarette smoking which play a key role for smoking initiation and becoming established smokers [7].

A prime aim of the Tobacco Control Act is to raise awareness on the health risks of tobacco use in the communities [8]. The awareness programmes on the health risks of smoking among young adults would help to reduce the risk of dying from a smoking-related illness and at the same time discourage smokers to quit smoking [9]. Understanding young adults’ perceptions on the health risks and the benefits of cigarette smoking is very essential. Here, perceived risk and benefits would mean subjective judgments that include the probability of occurrence of certain risks/negative outcomes and how an individual would concern with the consequences [10]. The meaning of risks varies from individual to individual and is influenced by social and culture structure of a community [10]. Hence, conducting a research on risk perception of smoking is crucial for it would even help to develop effective anti-smoking messages [11].

The perceived risks and benefits of cigarette smoking is one of the factors associated with smoking initiation among adolescents and young adults [12–14]. A community based survey from Nepal, for an example, showed that the adolescents who perceived benefits of smoking and did not think of health risks were at the risk of smoking initiation [12]. Next, the young adults who did not think smoking harmful to their health were more likely to be smokers than those who perceived health risks of smoking [5, 14]. The study also revealed that the young adult smokers did not believe in addictive nature of smoking [5]. Thus, understanding the relationship between risk perception and benefits of smoking is crucial for developing effective tobacco control programmes. It is also considered to be the first step towards behavioural change from risk-taking to safer behaviour. Perceptions of smoking-related health risks and benefits regarding young adults’ smoking behaviour have not been adequately studied in low-income countries like Nepal. Therefore, the current study has aimed to examine perceived health risks and benefits of cigarette smoking between young adults who smoke vs. don’t smoke.

## Methods

### Study design and setting

A cross-sectional study was conducted from August to September 2013 in Kathmandu Metropolitan, Nepal. There were two private medical colleges affiliated to Kathmandu University; three public health and seven management colleges affiliated to Pokhara University in Kathmandu Metropolis. These two medical colleges enrol 300 students while three public health colleges and seven management colleges enrol 120 and 560 students respectively each year. A list of private colleges was prepared with the permission and support from the concerned authorities of the universities. One medical college, two public health colleges and one management college were selected conveniently according to the study permission granted by the principals of those colleges. Therefore, we performed convenient sampling strategy to recruit the participants.

### Study population

The inclusion criteria for the study sample were: regular college students; both male and female; age between 18 and 24 years; enrolled in bachelor in medicine and surgery (MBBS), bachelor of public health (BPH) and bachelor of business administration (BBA) during the academic year 2013 in the colleges mentioned above; and their willingness to participate in the study. The study population represented from different parts of the country and belonged to different castes and ethnic groups. These students were enrolled in the study because they just entered into the college life after completion of their 12 years’ school education. They were also within vulnerable age-group: in-between adolescents and adults. However, with the right guidance, they could play key roles in tobacco control efforts [15].

### Sample size

The sample size was estimated based on the prevalence of smokers (*p* = 16 %) among young adults in Kathmandu with a required allowable error of 4 % and a 95 % confidence interval [5]. The approach yielded a sample size of 323 young adults. Overall response rate was 97.5 % i.e. 315 young adults.

### Measures

The semi-structured questionnaire contained three major sections: a) background variables or individual information; b) smoking behaviour; c) perceptions on smoking-related risks and benefits. The questionnaire was adopted from the Global Youth Tobacco Survey 2011 and from the perceived benefits and risks items from Dalton et al. [16, 17]. Previously, Dalton et al. measured positive and negative outcome expectations instead of perceived benefits and risks items [16]. Moreover, positive outcome expectation described individual sensory satisfaction from smoking and negative outcome expectation described health consequences of smoking [16]. The original questionnaire was modified to fit into the Nepalese context and pretested among public health and management students at private colleges affiliated to Purbanchal University, Nepal. However, the questionnaire was not translated into Nepalese version as the medium of teaching was English in all those selected colleges.

### Study variables and their definition

- Individual information
  Collection of information related to age, sex, and faculties.
- Smoking behaviour
  Ever smoker:
  The respondents who had not smoked cigarette in last 30 days prior to survey but had tried in the past (even a puff) was defined as ever smoker.
  Current smoking:
  The respondents who had smoked cigarette in last 30 days (even a puff) prior to the survey was defined as current smoking.
  Intention to smoke:
  The respondents who had planned to smoke cigarette in next 6 months is defined as intention to smoke [18].
- Perceived benefits of smoking items
  Perceived benefits of smoking were measured by asking questions in a hypothetical scenario: *Imagine you are planning to initiate cigarette smoking, do you believe in following benefits of smoking?* i. I believe smoking is enjoyable; ii. I believe smoking helps me to deal with problems or stress; iii. I believe smoking helps to stay thin; iv. I believe smoking helps me to feel more comfortable at gathering with friends; v. I believe smoking is relaxing; vi. I believe smoking would make me look more mature; and vii. I believe smoking gives me something to do when I am bored [16]. The respondents answered either ‘agree’ or ‘disagree’. The Cronbach’s alpha, which measures internal consistency was 0.78 and intra-class correlation coefficient (ICC) was 0.3.
- Perceived health risks of smoking items
  Perceived health risks of smoking were measured by asking questions in a hypothetical scenario: *Imagine you are planning to initiate cigarette smoking, do you believe in following health risks of smoking?* i. I believe smoking would make my teeth yellow; ii. I believe smoking would make my nails yellow; iii. If I started smoking regularly, I think it would be very hard for me to stop; v. I believe smoking would give me bad breath; and vi. I think smoking would be bad for my health [16]. The respondents answered either ‘agree’ or ‘disagree’. The Cronbach’s alpha and ICC was 0.61 and 0.18 respectively.

### Data collection

The anonymous, self-administrated English version questionnaire was used for data collection. Permission from the principals of respective colleges was taken prior to conduct the study. Research team explained the objectives of the study to the students before data collection. Data were collected by four trained medical and public health graduates. They distributed set of questionnaires to the students who met the inclusion criteria among those who were present in the classroom. It took around 10–15 min to complete a questionnaire.

### Data analysis

Data analysis was done using SPSS 17.0 version software. Percentage and mean (standard deviation) were computed to describe characteristics of respondents. Hazard Ratio (HR) was computed to estimate risk of current smoking behaviour with independent variables (sex, faculty and perceived risk and benefits of smoking) using Cox Proportion Hazard Regression Models with constant time at risk [19]. Individual variables (Sex and faculty) were analyzed in bi-variate analysis. Then, age, sex and faculty associated with current smoking behaviour were considered as confounders and treated as controlling variables for Cox Proportional Hazard Regression Models. The Kaplan-Meier analysis was used to estimate the mean age of smoking initiation in male and female respondents. The confidence limit with 95 % CI was set to assess the proportion and hazard risks. *P* value < 0.05 was considered as statistically significant.

### Ethical considerations

The ethical clearance was obtained from Kathmandu Medical College Ethical Committee. Permission was sought from the principals of each selected college and written informed consent was taken from the students. The objectives of the study and confidentiality of the information were explained and assured to both the principals and the students prior to data collection. The students’ participation was voluntary and requested to provide correct information.

## Results

### Respondent characteristics

**Fig. 1:**
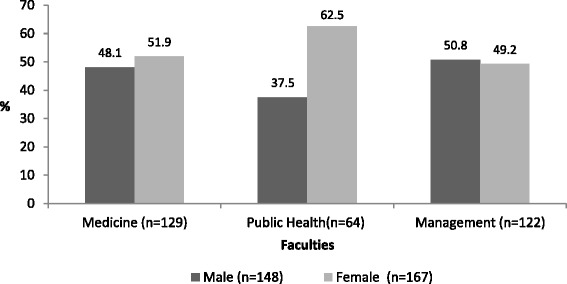
Sex and faculty wise distribution of students of each faculty

Figure 1 explains the characteristics of 315 respondents. Of the total respondents, 41 % were studying medicine (male = 48.1 %); 38.7 % studying management (male = 37.5 %); and 20 % studying public health (male =50.8 %). The sex ratio was 1.12 female per male (53 % vs. 47 %). Mean age of respondents was 19.12 (SD = 1.08) years.

### Smoking prevalence among respondents

**Fig. 2:**
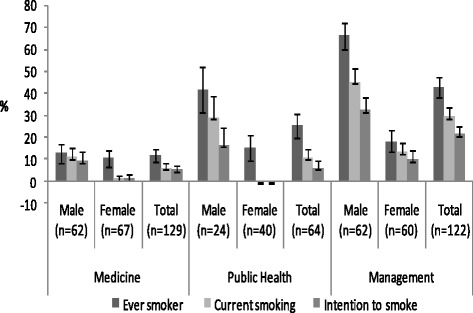
Sex and faculty wise proportion of ever smoker, current smoking and intention to smoke

Figure 2 describes the sex and faculty wise distribution of smoking prevalence. The prevalence of ever smoker was 26.4 % (95 % CI: 21.5; 31.5), of which the male and female ever smoker prevalence was 39.9 % (95 % CI: 31.9; 47.8) and 14.3 % (95 % CI: 9.01; 19.7) respectively. Likewise, the prevalence of current smoking was 16.2 % (95 % CI: 12.3–31.5) with the prevalence of current smoking for male and female was 28.4 % (95 % CI: 21.1; 35.6) and 5.38 % (95 % CI: 1.9; 3.6) respectively. The prevalence of intention to smoke was 11.7 % (95 % CI: 8.2; 15.3), where the prevalence of intention to smoke among male was 20.2 % (95 % CI: 13.7; 26.8) and among female it was 4.19 % (1.2; 7.3).

The risk of current smoking was higher among the male than the female (HR = 5.30; 95 % CI: 2.58; 10.89). Equally, the risk of intention to smoke was also higher among the male (HR = 4.89; 95 % CI: 2.12; 11.10). Next, faculty wise it was found that the risk of becoming current smoking was significantly higher being a management student (HR = 4.72; 95 % CI: 2.19; 10.20) than being a medical student. However, as a public health student, there was a non-significant higher risk of becoming current smoking than a medical student (HR = 1.75; 95 % CI: 0.64; 4.82).

**Fig. 3:**
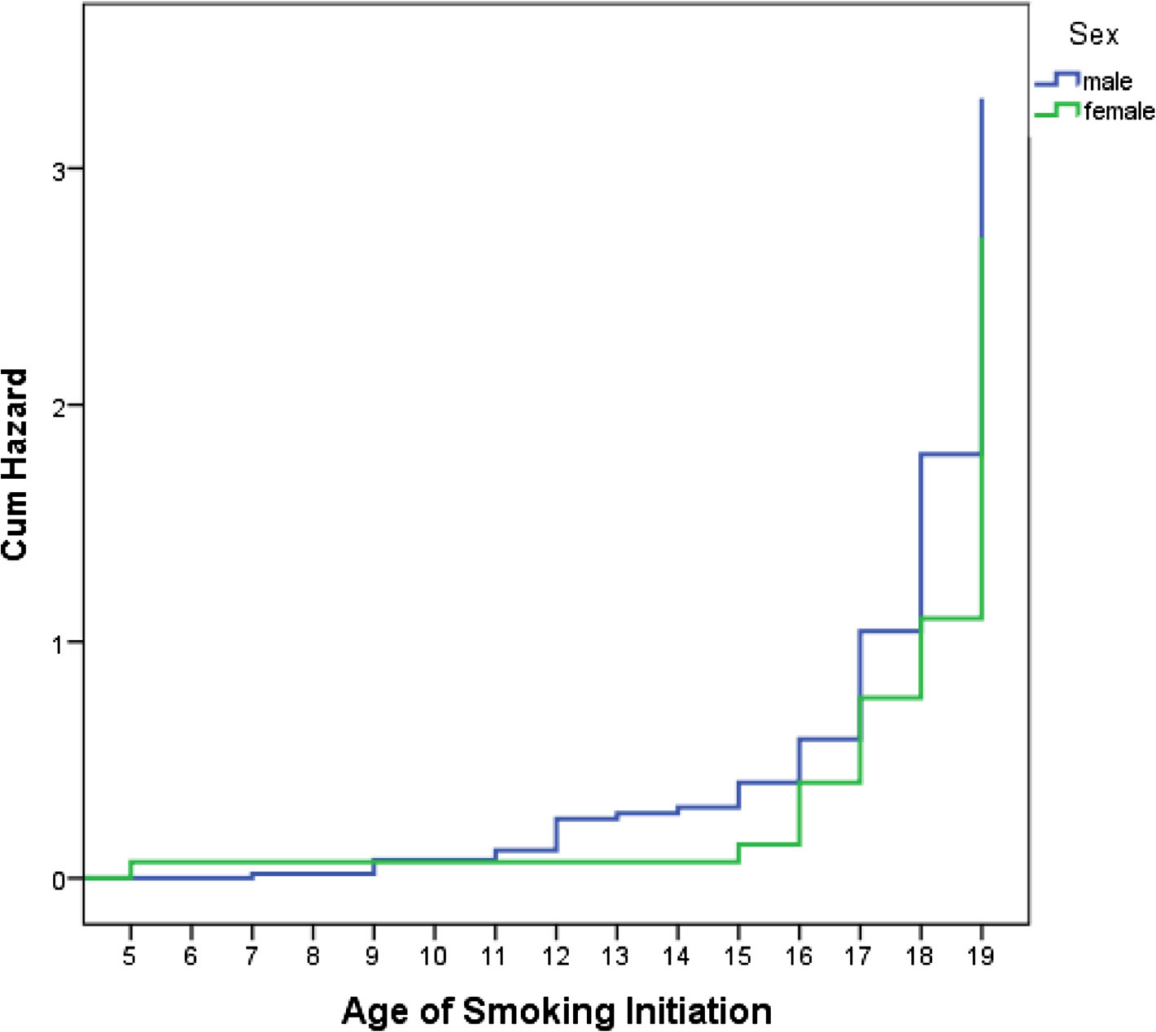
Cumulative hazard plot of age of smoking initiation by gender. There was no statistically significant difference in age of smoking initiation between male and female (log rank score 3.53, *P* = 0.06)

Cumulative hazard plot revealed that the majority of respondents had their first cigarettes between 11 and 19 years (Figure 3). For male, the risk of smoking initiation was found elevating after 12 years of age and gradually increased until 16 years, and then rapid growth until 19 years. The risk of smoking initiation among female was found constant up to the age of 16 years and then rapid growth until 19 years. The mean age of smoking initiation was 16.6 (95 % CI: 15.8–17.3) and 17.7 (95 % CI: 15.8–19.4) years for male and female respectively.

### Relationship between perceived benefits and current smoking behaviour of young adults: Cox proportional hazard ratio

Table 1 describes the perceived benefit items associated with the current smoking behaviour after controlling age, sex, and study faculty. The risk of becoming current smoking was higher among the students/respondents who believed in smoking would be relaxing (HR = 6.95, 95 % CI: 3.60; 13.43). Likewise, the risks of current smoking behaviour increased with those who believed that smoking is enjoyable (HR = 4.74, 2.58; 8.72); feel more comfortable at gathering with friends (HR = 4.29, 2.33; 7.92); smoking would give them something to do when they were bored (HR = 3.42, 1.91; 6.13); and would help to deal with problems or stress (HR = 3.19; 1.76; 5.79). The study results also showed that there was a non- significant increased risk of becoming current smoking among those respondents who believed smoking would help them to stay thin (HR = 1.29, 0.68; 2.46); and smoking would make them look more mature (HR = 1.65, 0.77; 3.53).

**Table 1:** Relationship between perceived benefits and current smoking behaviour among young adults (18–24 years)

Items	Percentage of young adults who believed in the benefits of smoking		Adjusted hazard ratio (95 % CI)^a^	*p* value^b^
	Current smoking (*n* = 51)	Non-smoking (*n* = 264)		
I believe I would enjoy smoking	30(58.8)	13(5.0)	**4.74(2.58;8.72)**	***P*** **< 0.001**
I believe smoking would help me to deal with problems or stress	26(51.0)	18(6.8)	**3.19(1.76;5.79)**	***P*** **< 0.001**
I believe smoking would help me to stay thin	13(25.5)	30(11.5)	1.29(0.68;2.46)	0.44
I believe smoking would help me to feel more comfortable at gathering with friends	32(62.7)	23(8.7)	**4.29(2.33;7.92)**	***P*** **< 0.001**
I believe smoking would be relaxing	38(74.5)	26(9.9)	**6.95(3.60;13.43)**	***P*** **< 0.001**
I believe smoking would make me look more mature	8(15.7)	22(8.4)	1.65(0.77;3.53)	0.19
I believe smoking would give me something to do when I m bored	25(49.0)	20(7.6)	**3.42(1.91;6.13)**	***P*** **< 0.001**

^a^Hazard ratios (HR) adjusted for age, sex, and faculty ^b^bold indicates results are significant

### Relationship between perceived health risks and current smoking behaviour of young adults: Cox proportional hazard ratio

Table 2 describes the perceived health items associated with the current smoking behaviour after controlling age, sex, and study faculty. The young adults who believed that smoking would make yellow teeth (HR = 0.53, 0.30; 0.94) and yellow nail (HR = 0.53, 0.29; 0.95) were at lower risk of becoming current smoking. Likewise, the respondents who perceived smoking was bad to their health were also at lower risk of becoming a current smoking (HR = 0.45, 0.2; 0.98). Similarly, the study showed a non-significant higher risk of becoming a current smoking who believed that stopping smoking once initiated was hard (HR = 1.20, 0.54; 2.67). The risk of having a current smoking had a non-significant inverse association with the perceived health risk i.e. young adults who believed that the smoking would give them bad breath 0.45 (0.2; 1.00).

**Table 2:** Relationship between perceived health risks and current smoking behaviour among young adults (18–24 years)

Items	Percentage of young adults who believed in the health risks of smoking		Adjusted hazard ratio (95 % CI)^a^	*p* value^b^
	Non-smoking (*n* = 264)	Current smoking (*n* = 51)		
I believe smoking would make my teeth yellow	190(72.8)	28(54.9)	**0.53(0.30;0.94)**	**0.031**
I believe smoking would make my nails yellow	137(52.5)	17(33.3)	**0.53(0.29;0.95)**	**0.034**
If I started smoking regularly, I believe it would be very hard for me to stop	226(85.9)	44(86.3)	1.20(0.54;2.67)	0.65
I believe smoking would give me bad breath	255(97.0)	44(86.3)	0.45(0.2;1.00)	0.44
I believe smoking would be bad for my health	258(98.1)	43(84.3)	**0.45(0.21;0.98)**	**0.04**

^a^Hazard ratios (HR) adjusted for age, sex, and faculty, ^b^bold indicates results are significant

## Discussion

This study has illustrated the smoking is common among Nepalese college students and how young adults have had the perceived health risks and the benefits of smoking. Even this study has tried to explore risk associated with smoking behaviour among male and female along with their faculties. Further, the study has shown the association of the risks of being a current smoking according to their perceptions on health risks and the benefits of smoking among young adults.

The study has also revealed that the current smoking prevalence and the proportion of intension to smoke among medical students was significantly lower than the non-medical students (management student) and comparable with similar studies [6, 20–22]. Further, this study showed that the management students were at greater risk of becoming smokers than the medical students. Several studies including in Nepal demonstrated that the smoking prevalence was higher among non-medical college students than the medical students [6, 21, 23]. A similar study in India also showed that exposure rate of tobacco was significantly higher among non-medical groups than medical groups (31 % vs. 10 %) [23]. A study from Ukraine revealed that the young adults who were rarely exposed to tobacco were at lower risk of smoking initiation [24]. Though this study did not measure the respondents’ exposure rate of tobacco, it might be one of the possible reasons for high prevalence of smoking as well as for higher risk of becoming smokers [25].

Besides exposure to tobacco, young adults/college students are one of the target groups of tobacco companies because of the following reasons: they can easily progress from “experimenter” to “established smokers” by an important increase in consumption; they face multiple life transitions that provide opportunities for adaptation and solidification of smoking as a regular part of new activities; and stresses of these life transitions invite them to initiate to smoke cigarettes for the drug effects of nicotine [26].

The analysis further revealed that overall and sex-wise prevalence of current smoking and intention to smoke was significantly low among the medical and the public health students than non-medical students which is consistent with the previous studies [5, 20, 22]. International review of literatures revealed that there was variation in smoking prevalence rate among medical students across the countries [22]. The prevalence rate was lower among female medical students than their male counterparts in the same medical college [22]. Next, this study demonstrated that the male young adults were five times more likely to have risk of being a current smoking than their female counterparts. Similarly, Ukrainian young adult male were more likely to initiate smoking when they reported low knowledge of tobacco related diseases (HR > 1) [24]. Two cross-sectional studies conducted in Nepal also explored that the male were more likely to be established smokers [6, 27]. Thus, gender differences is one of the most important risk factor predictors of smoking behaviours among Asian population [28].

Saudi-Arabian college students who smoked cigarettes had significantly different knowledge about the harmful effects of smoking [29]. A study by Aryal et al. demonstrated the young adult smokers were less aware of risks and health consequences of smoking [5]. An American study explained the same that young adult smokers did not have understanding about the risks associated with smoking [30]. A Ukrainian study demonstrated that male young adults who had lower knowledge about harmful effects of smoking were at risk of smoking initiation [24]. Likewise, the same Ukrainian study revealed that there was inverse relationship between tobacco related knowledge and current established smoking among male and female [24]. Recent community based study from Nepal revealed that the perceived social benefits and an addiction risk of smoking and smoking susceptibility was positively associated among adolescents [12]. Further, the perceived short-term physical risks of smoking are inversely related with smoking susceptibility [12]. However, such a research has not been conducted yet among college students, but this study has provided similar findings after controlling age, sex, and faculty.

The findings of this study show that the majority of the young adults had their first cigarettes during adolescence. Age of smoking initiation was lower among male than female which is consistent with previous study [31]. There are several socio-demographic and family factors as well as childhood environment factors including risk perception of smoking, which influence them to initiate smoking [12, 32]. Next reason is the lack of knowledge on smoking consequences, and having smoking related positive beliefs which lead them to initiate smoking [33]. According to GYTS 2011, only 51.3 % adolescents informed that their teachers discussed about the reasons for smoking and 67.8 % students had been taught in the class about the effects of smoking [34]. These figures indicate a large number of adolescents are still unaware of harmful effects of smoking. It is an urgent need to identify the reasons why larger percentage of adolescents was still unexposed to discussion on tobacco in classrooms as well as teaching about health effects of tobacco. Such adolescents might be at a greater risk of being future smokers due to the lack of adequate knowledge on harmful effects of tobacco use. Therefore, it is necessary to conduct researches on tobacco on the aforementioned issues by applying rigorous methodology to obtain reliable and valid information for effective intervention [35].

Finally, though the findings of this study mainly focused on the perceived risk and the benefits of cigarette smoking but this study also determined that the sex and the academic faculty were also important factors for current smoking behaviour of young adults. Thus, multi-component interventions are essential for effective tobacco control program.

### Limitations of the study

Despite the findings of this study provide important information on young adults’ smoking behaviours, the study is not free from its limitations. First, this study adopted non-random sampling, thus the findings cannot be generalized to all young adults college students of other part of the country. It is recommended to take large and representative sample of college students by applying nested or cluster sampling techniques. Second, a sample size (number of smokers) is not large enough to perform faculty-wise Cox-regression analysis. It can also be recommended that the sample size need to be calculated based on power analysis in future study. Third, the data were gathered from self-reported questionnaire and collected only once. Thus, causal relationship between perceived items and smoking could not be established. Thus, the longitudinal study is essential to establish cause-effect relationship. Finally, this study did not include the questions on smoking related variables like socio-demographic factors except age and sex; environmental factors; and behavioural factors. Therefore, the future study should incorporate above mentioned factors to understand its affects on perceived risks and benefits.

## Conclusions

Proportion of smoking was higher among young adult male as well as among management students. Furthermore, young adults who perceived the benefits of smoking were at risk of being a current smoking and perceived health risks were less likely to be a non-smoking young adult. Therefore, to discourage young adults from smoking, future intervention programmes should focus communicating not only health risks but also counteract perceptions of benefits related to smoking.

## References

[1] Alwan A (2011). Global Status Report on Noncommunicable Diseases 2010.

[2] World Health Organization (2011). Noncommunicable Diseases Country Profiles 2011.

[3] Ministry of Health and Population [Nepal] (2014). Noncommunicable Diseases Risk Factors: STEPS Survey Nepal 2013.

[4] Ministry of Health and Population[Nepal] (2012). Global Health Students Profesional Survey:2011.

[5] Aryal UR, Lohani S (2011). Perceived risk of cigarette smoking among college students. JNHRC.

[6] Binu V, Subba S, Menezes R, Kumar G, Ninan J, Rana M (2010). Smoking among Nepali youth– prevalence and predictors. Asian Pac J Cancer Prev.

[7] Tyas SL, Pederson LL (1998). Psychosocial factors related to adolescent smoking: a critical review of the literature. Tob Control.

[8] Ministry of Health and Population[Nepal] (2011). Tobacco Control and Regulation Act 2011.

[9] Yang J, Hammond D, Driezen P, Fong GT, Jiang Y (2010). Health knowledge and perception of risks among Chinese smokers and non-smokers: findings from the Wave 1 ITC China Survey. Tob Control.

[10] Weinstein ND (1980). Unrealistic optimism about future life events. J Pers Soc Psychol.

[11] Helweg-Larsen M, Nielsen GA (2009). Smoking cross-culturally: risk perceptions among young adults in Denmark and the United States. Psychol Health.

[12] Aryal UR, Petzold M, Krettek A (2013). Perceived risks and benefits of cigarette smoking among Nepalese adolescents: a population-based cross-sectional study. BMC Public Health.

[13] Song AV, Morrell HE, Cornell JL, Ramos ME, Biehl M, Kropp RY, Halpern-Felsher BL (2009). Perceptions of smoking-related risks and benefits as predictors of adolescent smoking initiation. Am J Public Health.

[14] Seigers DK, Terry CP (2011). Perceptions of risk among college smokers: relationships to smoking status. Addic Res Theory.

[15] Lenz BK (2004). Tobacco, depression, and lifestyle choices in the pivotal early college years. J Am Coll Health.

[16] Dalton MA, Sargent JD, Beach ML, Bernhardt AM, Stevens M (1999). Positive and negative outcome expectations of smoking: implications for prevention. Prev Med.

[17] Global Youth Tobacco Survey (GYTS) Core Questionnaire. 2011. [http://nccd.cdc.gov/gtssdata/Ancillary/DownloadAttachment.aspx?ID=33] (Accessed on November 15, 2012).

[18] Halpern-Felsher BL, Biehl M, Kropp RY, Rubinstein ML (2004). Perceived risks and benefits of smoking: differences among adolescents with different smoking experiences and intentions. Prev Med.

[19] Barros AJ, Hirakata VN (2003). Alternatives for logistic regression in cross-sectional studies: an empirical comparison of models that directly estimate the prevalence ratio. BMC Med Res Methodol.

[20] Sreeramareddy CT, Suri S, Menezes RG, Kumar H, Rahman M, Islam MR, Pereira XV, Shah M, Sathian B, Shetty U (2010). Self-reported tobacco smoking practices among medical students and their perceptions towards training about tobacco smoking in medical curricula: a cross-sectional, questionnaire survey in Malaysia, India, Pakistan, Nepal, and Bangladesh. Subst Abuse Treat Prev Policy.

[21] Zhu T, Feng B, Wong S, Choi W, Zhu SH (2004). A comparison of smoking behaviors among medical and other college students in China. Health Promot Int.

[22] Smith D, Leggat P (2007). An international review of tobacco smoking among medical students. J Postgrad Med.

[23] Chatterjee T, Haldar D, Mallik S, Sarkar G, Das S, Lahiri S (2011). A study on habits of tobacco use among medical and non-medical students of Kolkata. Lung India.

[24] Andreeva TI, Krasovsky KS, Semenova DS (2007). Correlates of smoking initiation among young adults in Ukraine: a cross-sectional study. BMC Public Health.

[25] Rigotti NA, Moran SE, Wechsler H (2005). US college students’ exposure to tobacco promotions: prevalence and association with tobacco use. Am J Public Health.

[26] Ling PM, Glantz SA (2002). Why and how the tobacco industry sells cigarettes to young adults: evidence from industry documents. Am J Public Health.

[27] Sreeramareddy CT, Kishore P, Paudel J, Menezes RG (2008). Prevalence and correlates of tobacco use amongst junior collegiates in twin cities of western Nepal: a cross-sectional, questionnaire-based survey. BMC Public Health.

[28] Tsai Y-W, Tsai T-I, Yang C-L, Kuo KN (2008). Gender differences in smoking behaviors in an Asian population. J Women Health.

[29] Al-Mohamed H, Amin T (2010). Pattern and prevalence of smoking among students at King Faisal University, Al Hassa, Saudi Arabia. East Mediterr Health J.

[30] Murphy-Hoefer R, Alder S, Higbee C (2004). Perceptions about cigarette smoking and risks among college students. Nicotine Tob Res.

[31] Verlato G, Melotti R, Corsico AG, Bugiani M, Carrozzi L, Marinoni A (2006). Time trends in smoking habits among Italian young adults. Respir Med.

[32] Aryal UR, Petzold M, Bondjers G, Krettek A (2014). Correlates of smoking susceptibility among adolescents in a peri-urban area of Nepal: a population-based cross-sectional study in the Jhaukhel-Duwakot Health Demographic Surveillance Site. Glob Health Action.

[33] Cosci F, Zagà V, Bertoli G, Campiotti A (2012). Significant others, knowledge, and belief on smoking as factors associated with tobacco use in Italian adolescents. ISRN Addict.

[34] Ministry of Health and Population[Nepal] (2012). Global Youth Tobacco Survey:2011.

[35] World Health Organization (2010). Guidelines for Implementation of Article 12 of the WHO Framework Convention on Tobacco Control (Education, Communication, Training and Public Awareness).

